# Explaining *in-vitro* to *in-vivo* efficacy correlations in oncology pre-clinical development via a semi-mechanistic mathematical model

**DOI:** 10.1007/s10928-023-09891-7

**Published:** 2023-11-06

**Authors:** Heinrich J. Huber, Hitesh B. Mistry

**Affiliations:** 1 Drug Discovery Sciences, Boehringer Ingelheim RCV GmbH & Co KG, Dr. Boehringer-Gasse 5-11, Vienna, 1120 Austria; 2 Department, SEDA Pharmaceutical Development Services, Oakfield Road Cheadle Royal Business Park, Cheadle, SK8 3GX United Kingdom

**Keywords:** IVIVC, PKPD modelling, Tumour growth inhibition, Xenograft studies

## Abstract

*In-vitro* to *in-vivo* correlations (IVIVC), relating *in-vitro* parameters like *IC*50 to *in-vivo* drug exposure in plasma and tumour growth, are widely used in oncology for experimental design and dose decisions. However, they lack a deeper understanding of the underlying mechanisms. Our paper therefore focuses on linking empirical IVIVC relations for small-molecule kinase inhibitors with a semi-mechanistic tumour-growth model. We develop an approach incorporating parameters like the compound’s peak-trough ratio (*PTR*), Hill coefficient of *in-vitro* dose-response curves, and xenograft-specific properties. This leads to formulas for determining efficacious doses for tumor stasis under linear pharmacokinetics equivalent to traditional empirical IVIVC relations, but enabling more systematic analysis. Our findings reveal that *in-vivo* xenograft-specific parameters, specifically the growth rate (*g*) and decay rate (*d*), along with the average exposure, are generally more significant determinants of tumor stasis and effective dose than the compound’s peak-trough ratio. However, as the Hill coefficient increases, the dependency of tumor stasis on the *PTR* becomes more pronounced, indicating that the compound is more influenced by its maximum or trough values rather than the average exposure. Furthermore, we discuss the translation of our method to predict population dose ranges in clinical studies and propose a resistance mechanism that solely relies on specific *in-vivo* xenograft parameters instead of *IC*50 exposure coverage. In summary, our study aims to provide a more mechanistic understanding of IVIVC relations, emphasizing the importance of xenograft-specific parameters and *PTR* on tumor stasis.

**Supplementary Information:**

The online version contains supplementary material available at 10.1007/s10928-023-09891-7.

## Introduction

Pharmacology research projects typically begin by investigating the mechanisms of drug action in simple systems, such as cultured cells in a dish, known as *in-vitro* experiments. Subsequently, these studies progress to explore more complex and living systems, including rodents, larger animals, and ultimately human subjects in clinical trials, known as *in-vivo* experiments. These investigations involve assessing the drug’s efficacy, its exposure (i.e., the amount of drug present in blood plasma) in the living system, and its tolerability for any potential unwanted side effects.

To facilitate clinical decision-making, it is crucial to establish the translatability of results from simple *in-vitro* systems to more complex *in-vivo* systems. This translatability is essential for interpreting study outcomes, designing experiments, and enabling studies in higher species [1]. In order to address this need, the use of *in-vitro* to *in-vivo* correlation (IVIVC) studies is necessary [2]. These studies, particularly focus on investigating drug efficacy, often establish a relationship between a specific aggregated *in-vitro* parameter and the required drug exposure in plasma, thus explaining a particular drug-induced effect.

In pre-clinical oncology studies, for instance, the *in-vivo* parameter of interest is tumour growth inhibition (TGI), which is evaluated through a xenograft study comparing the treated group to the control group at a specific time point. Similarly, the corresponding *in-vitro* parameter could be the IC50 value obtained from a 2-dimensional or 3-dimensional proliferation assay, or a biomarker assay that takes into account the *in-vivo* drug concentration.

Recently, the availability of systematic collections of public data for small therapeutic molecules has greatly facilitated IVIVC investigations. In a recent study [3], a diverse set of 164 small molecules representing different therapeutic indications, various modes of action (such as antagonists and partial agonists), and receptor types (including inhibitors of G-protein coupled receptors, ion channels, kinases, nuclear receptors, etc.) were examined. The researchers discovered that the ratio of the free plasma concentration (adjusted for plasma protein binding) to the *in-vitro* cell proliferation IC50 value followed a sigmoid curve, when correlated with the cumulative clinical efficacy of the compounds.

Above findings suggest that there exists a classical, logistic pharmacological exposure-response relationship *in-vivo*. The study further revealed that for 80% of the compounds, full efficacy was achieved when the aforementioned ratio fell between 0.007 and 8, with an average IC50 coverage of 0.32 of the free plasma concentration. In another oncology study with a stronger clinical focus, Goldstein and colleagues [4] analyzed 21 receptor tyrosine kinase inhibitors and 4 PARP inhibitor small-molecule-based cancer therapies. They found that for 76% of the compounds, the ratio of free plasma concentration to the reported IC50 value ranged from 0.4 to 4.

The latter study emphasizes the potential of leveraging *in-vitro* information and clinical pharmacokinetics (PK) for dose finding in oncology [1]. However, estimating doses based solely on *in-vivo* coverage of a specific *in-vitro* IC50 value can be challenging [5]. Even in pre-clinical settings, *in-vitro* proliferation *IC*50 values can vary significantly across different cell cultures when exposed to the same compound, spanning several orders of magnitude [6]. Nevertheless, such *IC*50 coverage laws by projected pharmacokinetic properties have been recently proposed as a scoring mechanism for compound selection and prioritisation in early compound design and optimisation [7].

The complexity of studying IVIVC is further increased in mouse xenograft studies, where different tumour cell lines are implanted to study tumour growth and drug-induced inhibition. In particular, the emergent properties of 3D tumour growth specific to the cell types used can introduce further variability in tumour response, incorporating features that are not present in *in-vitro* cell assays. These features may include *in-vivo* tumour drug exclusion, tumour cell-stroma interaction, and immune modulation of the tumour microenvironment. Furthermore, in clinical settings, factors such as high patient heterogeneity, toxicity limitations, and study and cohort biases contribute to additional uncertainties in dose-response predictions. Taken together, these factors underscore the challenges associated with translating *in-vitro* information to clinical dose determination in oncology. While *in-vitro* data and clinical PK provide valuable insights, it is important to consider the complexity and variability introduced by *in-vivo* and clinical settings when making dose predictions.

To partly address the heterogeneity observed in pre-clinical research, semi-mechanistic mathematical models that link pharmacokinetics (PK) to pharmacodynamics (PD) and tumour growth have been developed (referred to as PK/PD/TGI models) [8–11]. These models aim to capture the tumour evolution in mouse xenografts throughout a xenograft experiment by incorporating temporal PK profiles with various dosing schemes and *in-vitro* IC50 parameter values for cell growth or target engagement. Additionally, they incorporate xenograft-specific growth and drug response parameters to account for the 3D features mentioned earlier. These models have shown success in pre-clinical research.

Given the success of semi-mechanistic modelling in pre-clinical research, we were interested in exploring whether dosage decisions based on IVIVC relationships can also be understood using these models. Initially, our analysis will focus on situations with negligible absorption phases and Hill functions with a coefficient of 1 and will be subsequently extended to more general conditions. By leveraging semi-mechanistic modelling, we aim to gain insights into the relationship between PK, PD, tumour growth, and dosage decisions. This approach may provide a more comprehensive understanding of the complex interplay between drug exposure, *in-vitro* IC50 values, and tumour response

The structure of the paper is as follows. In the methods section, we will review the Mayneord-like model structure for linear radius growth (Sect. 2.1) and extend it to study drug action (Sect. 2.2). We will introduce a procedure to fit tumour growth data using IC50-normalized exposures, following the method proposed by [3] to establish an IVIVC. We will also provide an overview of the legacy data set used (Sect. 2.4) and define some nomenclature (Sect. 2.5).

In the results section, we will utilize our Boehringer Ingelheim legacy data of reversible and irreversible MAP kinase (MAPK) inhibitors to demonstrate that tumour growth inhibition of xenograft data can be fitted by *IC*50-normalized drug exposures or concentrations. This allows us to determine the necessary *IC*50 coverage for tumour stasis and the efficacious dose for a typical compound of that class, assuming linear pharmacokinetics.

We will then present the semi-mechanistic model in Sect. 3.2, which includes xenograft model specific parameters (growth rate (g) and decay rate (d)). This model will serve as the foundation for further discussions.

In Sect. 3.3, we will show that *IC*50-based coverage laws for efficacy are equivalent to our modeling formalism under certain PK assumptions, providing a more fundamental justification for the previously empirically determined IVIVC curves.

Utilizing these model-informed coverage laws for stasis in Sect. 3.4, we will find that xenograft parameters *g* and *d* are often more decisive determinants of tumour stasis compared to variations in the peak-trough ratio between different compounds with the same exposure and *IC*50 values.

Section 3.5 will explore two relaxations of our underlying pharmacokinetic (PK) assumptions. At first (Sect. 3.5.1) we included a non-negligible absorption phase into our PK, albeit under the assumption that absorption and elimination processes can be well separated in time. At second (Sect. 3.5.2), we considered cooperative effects in the compound’s efficacy (Hill coefficient 1 in the PD function). For such cases, our results will suggest that, as the Hill coefficient increases, the peak-trough ratio becomes a more crucial parameter for tumour stasis than the average concentration or exposure, indicating a shift away from simple exposure-drivenness towards a $c_{max}/c_{trough}$-driven realm. This also highlights the dependence of $c_{max}/c_{trough}$ or AUC-drivenness on individual cancer models.

In Sect. 3.6, we will use the model-informed coverage formula to explain variability in the empirical IVIVC curves, which, to our best knowledge, has not been yet mathematically thoroughly investigated.

Finally, in Sect. 3.7, we will expand our investigation to drug efficacy in a population characterized by *IC*50, *g*, and *d*. Through this, we will propose a resistance mechanism based on the *g*/*d* ratio in pre-clinical and clinical cohorts that is independent of drug efficacy and dose levels.

## Methods

### Analysis of tumour growth assuming a proliferating rim

In the further, we will focus on models of a specific class. These models use biological findings that tumour growth is driven by an outer layer of cancer cells that are growing exponentially while tumours having a necrotic core [12]. This approach is quite commonly used for studying tumour growth in xenograft studies and has been well described and tested [13–15].

The approach used in this paper is based on a model and its validation to rat sarcoma xenografts published by Mayneord in 1932 [16] where tumour radius increases linearly over time, and hence, tumour volume grows with third order. Therefore, we will denote these models as *Mayneord-like models* troughout the manuscript. We note that besides such linear radius growth models also other approaches have been pursued in the field[17, 18].^1^

We briefly describe the method of Mayneord [16] here for convenience. For simplicity, [16] assumed that tumour growth is driven by an outer layer of cells with a small, temporally constant, but finite thickness $\Delta r\ll r$ and that the tumour that can be mapped onto a spherical shape. The first assumptions reflects the fact that every tumour of radius *r* has a necrotic core absent of living cells. Hence, the total volume of the tumour with radius *r* is the sum of volume of rim and core,

1 $$\begin{aligned} V_{total} \, =\, V_{rim}\, +\, V_{core}\, = \frac{4}{3}\,\pi r^3 \, , \end{aligned}$$

with volume of the outer rim $V_{rim}$ given by

2 $$\begin{aligned} V_{rim}= & {} 4/3\,\pi \,\left( r^3 -\left( r-\Delta r\right) ^3\right) \nonumber \\= & {} 4\,\pi \ r^2\, \Delta r + o\left( \Delta r^2)\right) , \end{aligned}$$

whereby we can neglect the higher order terms of $\Delta r$ for $\Delta r\ll r$.

As typical in studying growth processes, we assume first order growth of the tumour volume with the proliferation rate $\alpha$, but confining us to proliferation to the cells in the outer rim. We therefore get

3 $$\begin{aligned} \frac{d V_{total} }{dt} = \alpha \, V_{rim} \,=\ 4\,\alpha \,\pi \,r^2\Delta r \, . \end{aligned}$$

We now study the evolution of the total tumour radius *r* instead of the total volume $dV_{total}$ in Eq. (1)

4 $$\begin{aligned} \frac{dr }{dt} = \frac{dr }{dV_{total}}\,\cdot \, \frac{dV_{total} }{dt} = \left( \frac{1}{4 \pi r^2 }\right) \,\frac{dV_{total} }{dt} \, . \end{aligned}$$

which by inserting Eq. (4) in Eq. (3) gives for the temporal change of the tumour radius,

5 $$\begin{aligned} \frac{dr }{dt} = \left( \frac{1}{4 \pi r^2 }\right) \,\left( 4\,\alpha \,\pi \,r^2\Delta r \right) = \alpha \,\Delta r\,:=\,g, \end{aligned}$$

with introducing the growth rate $g=\alpha \,\Delta r$ for convenience reasons and assuming the thickness of the proliferating rim $\Delta r$ constant over time. We hence obtain the following tumour grow for Mayneord-like models

6 $$\begin{aligned} r(t) = R_0 + g\,t \, , \end{aligned}$$

with the initial tumour radius $R_{0}$ as integration constant.

Therefore, based on the Eq. (6), it can be inferred that under the assumption of a small growing rim relative to the radius ($\Delta r\ll r$), the tumor radius can be assumed to growth linear with time and hence the tumour volume grows with third order.

### Semi-mechanistic model for studying preclinical *in-vivo* tumour growth and drug-induced decay

As demonstrated in the previous subsection, it is reasonable to assume approximate tumor growth by a linear change of the tumour radius over time in the presence of a large necrotic core. These Mayneord-like models have been recently extended by us and other researchers [5, 19, 20] to investigate the reduction of tumor growth through pharmacological intervention using a compound.

In this context, the pharmacological action depends on both the drug exposure in the system over time (i.e., pharmacokinetics) and the *in-vitro* potency. The measure of *in-vitro* potency, *IC*50, can be obtained from a conventional *in-vitro* experiment, such as a drug-induced cell growth inhibition assay.

The dose-response of such assays is typically described by a Hill function, $f(c_{plasma, free}(t))$,

7 $$\begin{aligned} f(c_{plasma, \,free}(t)) = \frac{c_{plasma, \,free}(t)^{hill}}{c_{plasma, \,free}(t)^{hill} + IC50^{hill} }. \end{aligned}$$

Here, $c_{plasma, free}(t)$ represents the concentration of the compound in the free plasma, *hill* denotes the Hill coefficient, and *IC*50 corresponds to the concentration at which half of the maximal effect is observed. In the *in-vivo* context, the concentration becomes a function of time and is derived from the output of a pharmacokinetic model.

Moreover, we assume that the *IC*50 values originate from *in-vitro* proliferation experiments with sustained drug exposure, representing an aggregated value of *in-vitro* anti-tumor drug efficacy over time. Additionally, our model implicitly assumes the validity of the free plasma drug hypothesis and does not explicitly incorporate time delays between the free plasma drug concentration, $c_{plasma, free}$, and the effect at the target site.

Besides the influence of *in-vitro* pharmacology property $f(c_{plasma, free})$ on tumour growth, further properties emerge when studying whole 3D tumours. Such properties would be the permeability of the tumour to the compound, the content of tumour cells vs. stroma, co-operative effects between the cells in the tumour and so forth. These effects are specific to the human tumour cells implanted in a mouse and to the mouse strain.

While these effects are very complex, experience in our pre-clinical research informs us that they can for practical purposes be lumped into one parameter (the decay parameter *d*). This parameter therefore links tumour reduction to *in-vitro* pharmacology and aforementioned emergent *in-vivo* effects. This approach is further motivated in Appendix 6.

Based on above reasoning, the approach of [5, 19, 20], studies tumour evolution over the course of a xenograft experiment with the starting radius $R_0$ as follows,

8 $$\begin{aligned} \frac{dr}{dt}& = &g \, - \, d\, f(c_{plasma, \,free}). \nonumber \\ R& = &R_0 + g\tau \, - d\,\int ^\tau _0 \, \frac{c_{plasma, \,free}(t)^{hill}}{c_{plasma, \,free}(t)^{hill} + IC50^{hill} } \, d\tau \,, \end{aligned}$$

with the decay rate *d* as motivated above, while *g* denotes the above motivated linear tumour growth rate over time.

Both, the tumor growth and decay rates, are intrinsic properties of the *in-vivo* 3D tumor and depend on the specific cell graft mouse model employed.^2^ This allows for their reuse in studying different compounds with a similar mode of action as demonstrated in Appendix 6. Furthermore, these rates are assumed to be independent of the specific *in-vitro* pharmacology [14, 19].

As concluding remark, is important to note that the model framework distinguishes between compound-specific *in-vitro* pharmacology effects and *in-vivo* specific xenograft information related to tumor growth and drug-induced decay.

### Calculation of tumour growth inhibition and IVIVC curves

Throughout the manuscript, tumour growth inhibition is given by the fraction of change of tumour volume during a treatment period and the change of tumour volume of untreated/sham treated controls over that same period ($V^{treated}_{tumour}$ and $V^{control}_{tumour}$, respectively). Hence, this gives us

9 $$\begin{aligned} TGI = 100*\left( 1 -\frac{V^{treated}_{tumour} (end) - V^{treated}_{tumour} (start)}{V^{control}_{tumour} (end) - V^{control}_{tumour} (start)}\right) \, . \end{aligned}$$

In the results section, we will fit the relationship between the free average unbound plasma concentration, free *in-vitro* *IC*50 and TGI by the following logistic curve,

10 $$\begin{aligned} TGI = \frac{TGI_{max}-TGI_{min}}{1+\left( PD_{inflex}/ \frac{c_{average,ub}}{IC50}\right) ^{hill_{exp}}}, \end{aligned}$$

or using the *area under curve* of the unbound concentration over time $c_{ub}(t)$ over a dosing interval $\tau$, $AUC_{ub}$

11 $$\begin{aligned} TGI = \frac{TGI_{max}-TGI_{min}}{1+\left( \tau *PD_{inflex}/ \frac{AUC_{ub}}{IC50}\right) ^{hill_{exp}}} . \end{aligned}$$

**Fig. 1:**
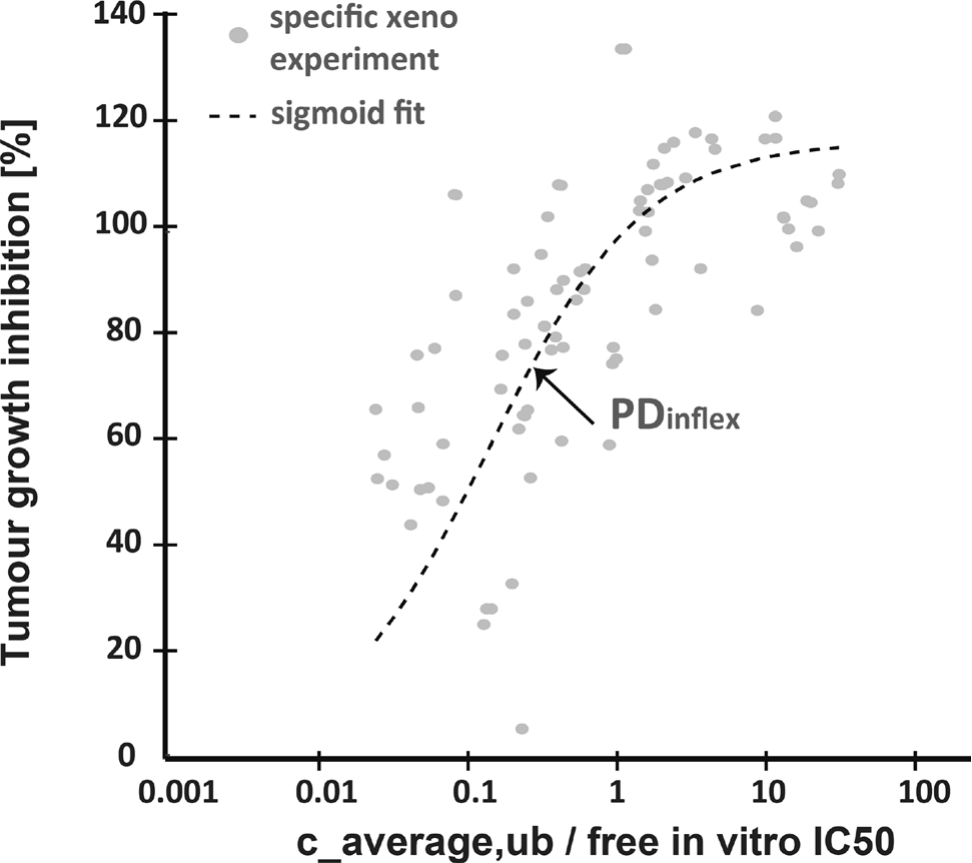
A pre-clinical IVIVC relationship between tumor growth inhibition and *IC*50-exposure coverage using data from Boehringer Ingelheim’s internal projects on receptor kinase inhibitors. A sigmoid fit curve is provided according to Eq. (10)

Here $PD_{inflex}$ is the inflection point, $hill_{exp}$ the Hill coefficient and $TGI_{max}$, $TGI_{min}$ the maximum and minimum TGI values of the fitting curve, respectively, as given in the figure captions of Fig. 1. The logistic curve is here purely heuristic and awaits a pharmacological interpretation.^3^

The empirical relation, Eq. (10), now allows us to estimate a required dose for a TGI of 100 percent, i.e. tumour stasis. We therefore have introduced a dose normalised unbound AUC ($AUC_{DN,ub}$). Assuming dose linearity, we use scaling to calculate a stasis dose $dose_{stasis}$,

12 $$\begin{aligned} TGI=100,\quad AUC{ub} = dose_{stasis}\,*\,AUC_{DN,ub}\, . \end{aligned}$$

Plugging (12) in Eq. (10) we obtain for the stasis dose $dose_{stasis}$

13 $$\begin{aligned} dose_{stasis} = \frac{PD_{inflex}}{\left( \frac{TGI_{max}-TGI_{min}}{100}-1\right) ^{1/hill_{exp}}} \, \frac{IC50}{AUC_{DN,ub}/ \tau }\, . \end{aligned}$$

### Illustration data

Data to illustrate modelling results in the manuscript were obtained from 86 mouse xenograft experiments (6 different xenografts, 12 reversible and covalent MAP kinase inhibitor compounds). Unbound *IC*50 values were taken from anti-cell proliferation measurements with 5 days incubation with the drug and ranged from 0.2-700 nM (median: 7.7). Total AUC ranged from 1.84-4141 nM*h (55), unbound fraction 0.04$-$2.3% (0.53) and study time $\ge$ 14 days of repeated *qd* or *bid* dosing. Control fractionation experiments with same daily dosing revealed no difference between *qd* and *bid* dosing for the specific set.

### Nomenclature

Throughout the manuscript, *in-vitro* *IC*50 and all *in-vivo* drug concentrations $c_{max}, c_{trough}, c_{average}$ resp. exposures (all *AUC*) were assumed as free drug fractions, hence being already corrected for plasma protein binding in the respective systems. Whenever of importance, this will be noted explicitly.

We further give information about the most commonly used terms in a Glossary, Sect. 4.

## Results

### Inferring efficacious doses from free plasma concentration, *in-vitro* efficacy and *in-vivo* tumour growth inhibition

It has been previously demonstrated that drug response *in-vivo* can be related to the plasma concentration of the free drug and *in-vitro* efficacy (as measured by *in-vitro* *IC*50 curves)^4^, [3]. Specifically, in an oncology context, a relation between tumor growth inhibition (TGI) and the fraction between the average free plasma concentration and free *IC*50 has been observed [3, 4]. We therefore aimed to replicate such findings with *in house data*.

For validation purposes, we utilized data from Boehringer Ingelheim’s internal compound library on MAPK inhibitors (see Fig. (1)). We plotted TGI against the fraction of the average free concentration over free *IC*50, where each dot represents a xenograft study involving 6 different xenografts and 12 compounds. We then fitted a curve to the logistic function as defined in Eq. (10) from the methods and obtained the following parameters: $PD_{inflex}$ = 0.25, $hill_{exp}$ = 0.89, $TGI_{max}$ = 125, $TGI_{min}$ = 0.

With this fitting parameters and assuming dose linearity, we were able calculate a stasis dose by Eq. (13) from the methods

14 $$\begin{aligned} dose_{stasis} = \frac{PD_{inflex}}{\left( \frac{TGI_{max}-TGI_{min}}{100}-1\right) ^{1/hill_{exp}}} \, \frac{IC50}{AUC_{DN,ub}/ \tau }\, , \end{aligned}$$

and obtained

15 $$\begin{aligned} dose_{stasis}\approx & {} 29.8 \, *\,\frac{IC50}{AUC_{DN,ub}} \end{aligned}$$

16 $$\begin{aligned} dose_{stasis}\approx & {} 1.24 \, *\,\frac{IC50}{c_{average, DN,ub}} \,, \end{aligned}$$

where we also have introduced the dose-normalised average concentration ($c_{average, DN,ub}$) and assumed a $\tau = 24$h dose interval, hence

17 $$\begin{aligned} c_{average, DN,ub}=AUC_{DN,ub}/\tau . \end{aligned}$$

The relations (15, 16) suggest that the required tumour stasis dose ($dose_{stasis}$) for an "average" compound of this IVIVC relation is proportional to the *IC*50 of the compound divided by either the dose normalised AUC ($AUC_{DN,ub}$) or dose-normalised average concentration ($c_{average, DN,ub}$).

We therefore have defined the inverse of the (compound and *in-vitro* model-specific) factors in Eqs. (15) and (16)

18 $$\begin{aligned} \frac{c_{average, DN,ub}}{IC50}\quad \text {and} \quad \frac{AUC_{DN,ub}}{IC50}\, , \end{aligned}$$

as *IC*50 coverage factors for $c_{average, DN,ub}$ and $AUC_{DN,ub}$, respectively.

These parameters will play a crucial role in our further investigations to study the required coverage of *IC*50 by the average concentration or by the time-integrated exposure over a dosing interval.^5^ As evident from Fig. (1) and Eq. (17), both factors exhibit a monotonous trend in relation to tumor growth inhibition (TGI).

### Relating empirical efficacious doses to mechanistic TGI Modelling

Above results related the inverse of the compound specific coverage factor (18) to tumour stasis. As this result is only grounded on empirical data, our objective was to ground these findings on a more mechanistic understanding of tumour growth.

Therefore, we asked if a similar relation could be established by using a standard PK/TGI mechanistic model. We therefore have used a model that has been shown to describe the underlying tumour growth mechanism quite well [13, 16, 19] and hence is heavily used in our Boehringer Ingelheim team for *in-vivo* xenograft study design. For the purpose of the paper, this model is therefore assumed as a fairly accurate coarse-grain description of the underlying tumour growth mechanism, hence being assumed as the “ground truth”.

The model was described in the methods Sect. (2.2). As shown there, we have used a combine model of tumour growth and drug-induced anti-tumour effect

19 $$\begin{aligned} dR\,= & {} \, \left[ g\, -\, d\,\left( \frac{c_{plasma, \,free}}{IC50 +c_{plasma, \,free} }\right) \,\right] dt \end{aligned}$$

20 $$\begin{aligned} R\,= & {} \, R_{0} + g\tau -\, d\,\int ^\tau _0\, \frac{c_{plasma, \,free}(t)}{IC50 +c_{plasma, \,free}(t) } \,dt, \end{aligned}$$

with the dosing interval $\tau$ and the initial tumour radius $R_{0}$. The TGI is then calculated from the volume at time $t=\tau$ via the relation ( 9), $Vol=4R^3\,\pi /3$ in the presence and absence of drug $(c_{plasma, free}(t))$ concentration, while *g* and *d* are *in-vivo* xenograft specific parameters as described in the methods.

### Derivation of model-informed plasma concentrations for tumour stasis

**Fig. 2:**
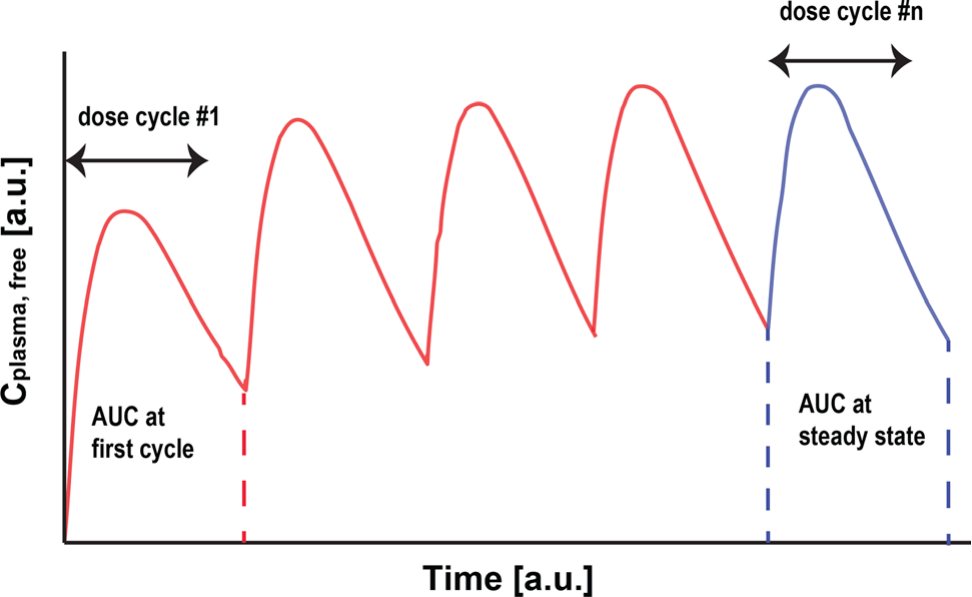
A typical pharmacokinetic behaviour for the free plasma concentration over time $c_{plasma, free }(t)$ is given for repeated dosing. Accumulation over repeated dosing cycles leads to a steady state behaviour of the daily exposure. The last dosing interval (in blue) has been assumed to be in steady state and to provide sufficient exposure to put the tumour to stasis (Color figure online)

To further the objective of the previous section, we now specifically asked for which *IC*50 coverage the above tumour model above would predict stasis. We therefore assumed that an equilibrium behaviour for the pharmacokinetics after multiple dosing has been achieved Fig. (2).

For tumour stasis, we then required that the compound exposure at these later cycles (area under the blue curve) is sufficient to halt tumour growth. This leads to the requirement

21 $$\begin{aligned} \int \,dR\,{\le }0 \, => \, g\,\tau /d \,{\le }\, \int ^\tau _0\, \frac{c_{plasma, \,free }(t)}{IC50 +c_{plasma, \,free}(t) }\, dt\,, \end{aligned}$$

where we have used the free plasma concentration $c_{plasma, free}(t)$ over time as the major surrogate for anti-tumour effect (know as free plasma concentration hypothesis).^6^

We next assumed a first order kinetic approximation of the free plasma concentration and again postulate dose linearity. We further assume a fast and hence negligible absorption phase and also assumed an Hill coefficient of 1. We later included some relaxations to these conditions in Sects. 3.5.1 and 3.5.2.

Hence, we have set

22 $$\begin{aligned} c_{plasma, \,free}(t) = c_{max}\,e^{-kt}\qquad k = \frac{1}{\tau }\,ln\left( \frac{c_{max}}{c_{trough}} \right) \end{aligned}$$

where we have denoted the maximum (“max”) and minimum (“trough”) free plasma concentration $c_{max}$ and $c_{trough}$. For brevity and by analogy reasons, we now define the effective AUC, $AUC_{effect}$

23 $$\begin{aligned} AUC_{effect} = \int ^{\tau }_0\, \frac{c_{max}\,e^{-kt}}{c_{max}\,e^{-kt} + IC50} \,dt \end{aligned}$$

and require a threshold for the stasis condition (21)

24 $$\begin{aligned} 0 = R-R_0 = g\tau \,-\,d\,AUC_{effect}\rightarrow \quad AUC_{effect}\, = \,\frac{g}{d}\cdot \tau \, . \end{aligned}$$

In the Appendix 7.1, we calculated Eq. (23) as

25 $$\begin{aligned} AUC_{effect} = \frac{\tau }{\text {ln}\left( PTR\right) }\text {ln}\left[ \frac{c_{max} + IC50}{c_{trough} + IC50} \right] \, . \end{aligned}$$

We have set here the *Peak-trough ratio* ($PTR=\frac{c_{max}}{c_{trough}}$) for convenience as this ratio has manageable variation within one compound class and can be estimated for typical small molecule compounds in oncology projects.

Setting the effective AUC in (B9) into the stasis condition Eq. (21), we were able to relate $c_{trough, stasis, ub}$, $c_{max, stasis, ub}$ and $c_{average, stasis, ub}$ with the compounds *PTR* and the tumour model specific parameters *g* and *d*. The detailed derivation is given in Appendix 7.2 and we obtain.

26 $$\begin{aligned} c_{trough, stasis, ub}& = &IC50 \frac{PTR^{g/d}-1}{PTR-PTR^{g/d}}\nonumber \\ c_{max, stasis, ub}& = &IC50\,\cdot \, PTR \frac{PTR^{g/d}-1}{PTR-PTR^{g/d}}\nonumber \\ c_{average, stasis, ub}& = &IC50\,\cdot \, \frac{PTR-1}{ln\left( PTR\right) } \frac{PTR^{g/d}-1}{PTR-PTR^{g/d}} . \end{aligned}$$

By this, we have established sufficiency condition of above concentrations to achieve tumour stasis when above assumptions are met.^7^

Finally, we used Eq. (26) to scale again the dose such that a stasis dose $dose_{stasis}$ is reached. We therefore replace $c_{average}$ by the total exposure over a dosing interval $\tau$ according Eq. (17) and assume again dose-linearity Eq. (12),

27 $$\begin{aligned} dose_{stasis} = \, \frac{PTR-1}{ln\left( PTR\right) } \frac{PTR^{g/d}-1}{PTR-PTR^{g/d}}\, \frac{IC50}{AUC_{DN,ub/ \tau }} \, . \end{aligned}$$

For convenience, we define the factor

28 $$\begin{aligned} MEF\left( PTR, g/d\right) \,:= & {} \,\frac{PTR-1}{ln\left( PTR\right) } \frac{PTR^{g/d}-1}{PTR-PTR^{g/d}} \, \end{aligned}$$

29 $$\begin{aligned}= & {} \frac{c_{average, stasis, ub}}{IC50}\,, \end{aligned}$$

which is dependent on the compound specific parameter *PTR* and the *in-vivo* xenograft specific parameters *g* and *d*. We will denote this factor as *model efficacy factor* which will give us further insights into the driver of this stasis dose and *IC*50 coverage by $c_{average, stasis, ub}$ in the further analysis.

By this, we can conclude that the stasis dose $dose_{stasis}$ can be reached either by optimising exposure over *IC*50 of the compound (right factor, *AUC-drivenness*) or by optimising $MEF\left( PTR, g/d\right) \,$ (*non AUC-drivenness*).

### Calculation of xenograft-model specific *IC*50 coverage for tumour stasis

Based on the defined coverage laws (26), we now aimed to systematically investigate their dependency on compound (*PTR*, *IC*50) and *in-vivo* xenograft specific parameters (*g* and *d*). In line with our internal data set, we used steady-state *PTR*s with 10-300 and a typical xenograft with a *g*/*d* ratio of 0.4 as observed in our pre-clinical studies. We then calculated necessary coverage for $c_{trough, stasis}$, $c_{max, stasis}$ and $c_{average, stasis}$ (26) for tumour stasis.

**Fig. 3:**
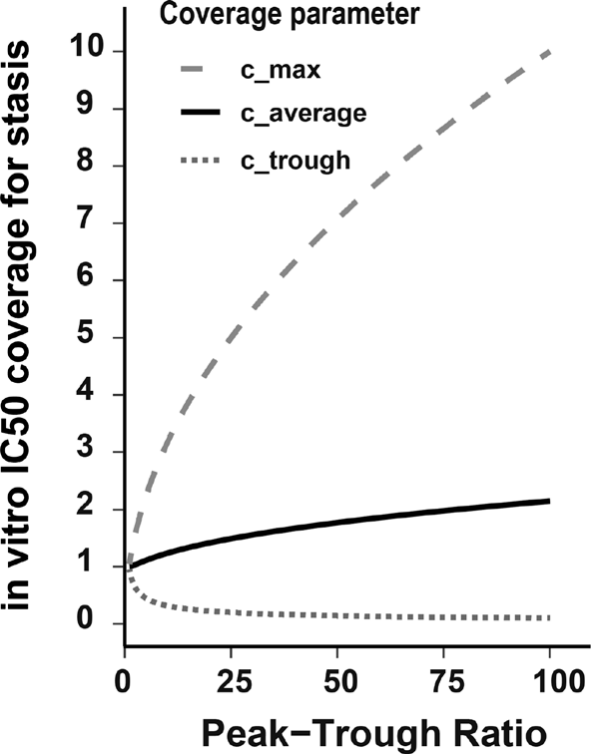
The ratio between the required *IC*50 coverage for tumour stasis for $c_{max}$ (dashed line), $c_{average}$ (solid line) and $c_{trough}$ (dotted line). We see that for typical pre-clinical *PTR*s of oncology compounds and a reasonable *g*/*d* ratio of 0.4, a $c_{average}$ coverage of about 2 is suggested. The high necessary $c_{max}$ coverage for larger *PTR* is to compensate for the less durable target engagement for such low terminal half-life compounds

Results are shown in Fig. 3 and indicate that for tumour stasis, *IC*50 coverage of the average unbound plasma concentration is essential, and less dramatically dependent from min-max variations (*PTR*). These results suggest “*AUC drivenness*” in xenografts and compounds with above properties.

**Fig. 4:**
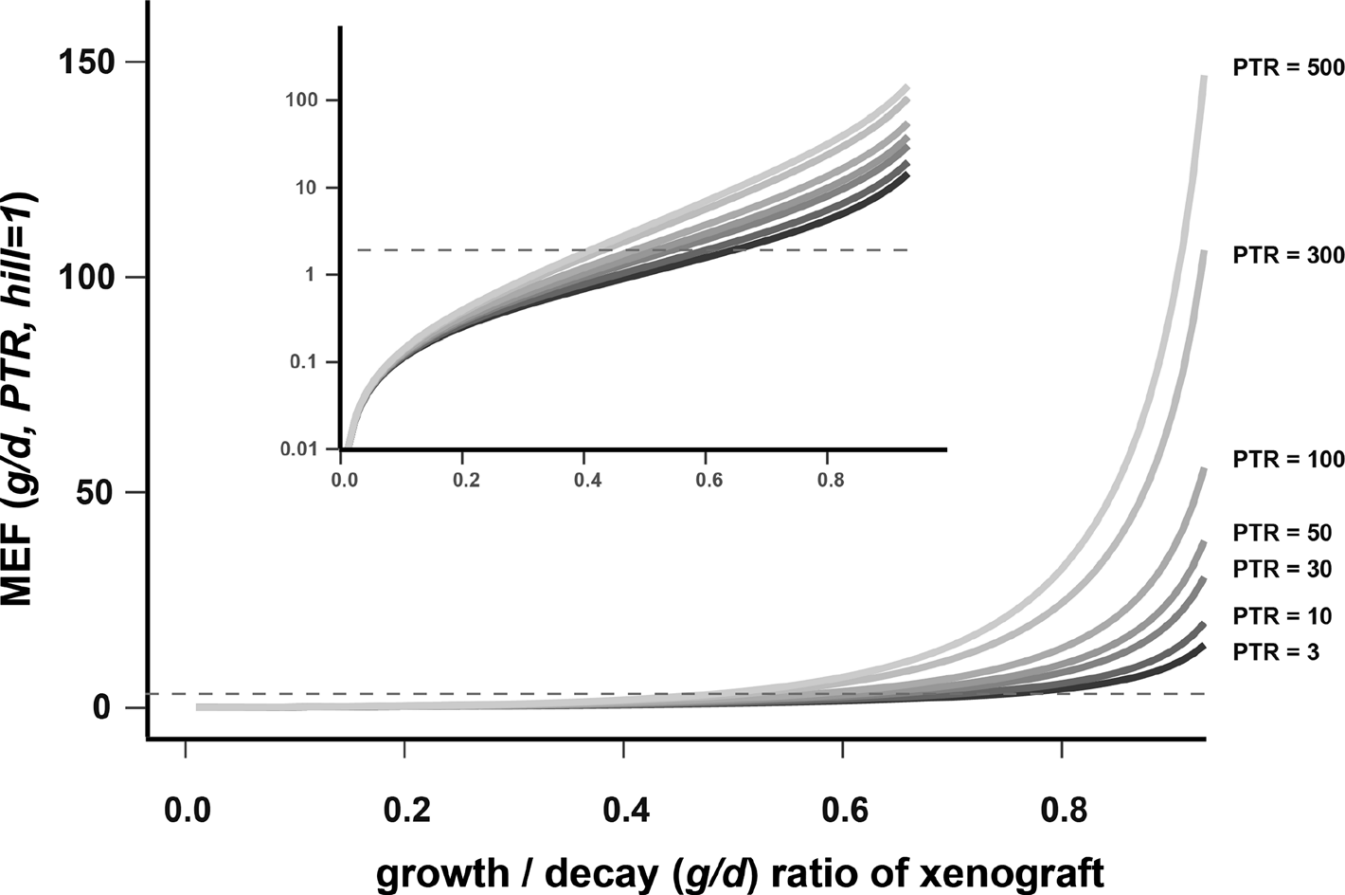
The model efficacy factor *MEF* given for the depicted Peak-Trough-ratios and different fractions of *g*/*d*. This factor indicates necessary *IC*50 coverage by $c_{average,ub, stasis}$ for stasis. The dashed line indicates MEF = 2. Inlet shows the same plot on a semi-logarithmic axis. PTRs were taken from compounds in Fig. 1. For model used in our research, *g*/*d* ratios for xenografts were in the range 0.3$-$0.7 (cf. Fig. 8 for our analysis of compounds.)

We then investigated how the coverage factor Eq. (28) changes for the different xenograft models characterised by given *g*/*d* ratio with *PTR* range of 40-150. We therefore plotted the model efficacy factor in Fig. 4. We observed that, when *g*/*d* ratios change between 0.3 and 0.7, these efficacy factor (and hence *IC*50 coverage factors) range from half *IC*50 coverage to about 10-fold coverage for our MAPK inhibitor compounds.

As expected, low g/d ratios (left edge), consistent with slow growing and relative sensitive xenograft models, required little *IC*50 coverage (on top of these cell-specific xenografts having potentially also a lower *in-vitro* *IC*50). In turn, when the growth rate increases relative to the decay rate $g/d\rightarrow 1$, high *IC*50 coverage is necessary for tumour stasis.

Interestingly, these coverage results were again mildly dependent for most assumed pre-clinical *PTR*s and *g*/*d* between 0.3 and 0.7, suggesting that *"AUC drivenness"* also mostly holds also for mildly sensitive to mildly resistant xenografts and compounds with reasonable max/trough variations.

Overall, these result indicate that the type of xenograft (besides influencing efficacy *per se*) influences the extent as to how efficacy is dependent on the compound’s *PTR*. We have thereby also obtained a more mechanistically grounded understanding of our empirical findings in (16), relating the fitting factor in (16) to xenograft- (*g*/*d*) and compound-specific (*PTR*) properties.

### Expanding the validity of the findings through relaxations of assumptions

For didactic purposed, we have assumed a negligible absorption phase and a Hill coefficient of 1 in our previous derivation. We now make two relaxations, whereby Relaxation 1 leaves the formalism unchanged, while Relaxation will provide further insights into the specific pharmacokinetic drivers of anti-tumor effect, once $hill>1$ (often denoted as *drug co-operativity*^8^).

#### Relaxation 1: application to situations with non-negligible absorption time

Above derivations were performed under the assumption of an exponential decay from $c_{max}$ to $c_{trough}$ at the entire dosage range. This may be considered as a crude assumption as orally (or other non i.v.) administrated compounds may show a non-negligible absorption phase.

Therefore, in Appendix 8, we demonstrated that above derivations still hold, when the absorption phase is not negligible and maximum concentration $c_{max}$ is achieved at a certain time $t_{max}$. We therefore had to postulate that absorption and elimination phase have different temporal dynamics (hence no flip-flop kinetics), which well holds for the investigate MAPK inhibitors reported here.^9^

#### Relaxation 2: tumour stasis conditions for compounds with $hill\ne 1$

An Hill coefficient different to one is often found in the *in-vitro* function Eq. (7) and is associated with positive or negative co-operative effects of the compound.

Therefore, we have repeated calculations of Sect. 3.3 for situations with $hill\ne 1$ as described by

30 $$\begin{aligned} R = R_{0} + g\tau -\, d\,\int ^\tau _0\, \frac{c_{plasma, \,free}(t)^{hill}}{IC50^{hill} +c_{plasma, \,free}(t)^{hill} } \,dt\, . \end{aligned}$$

Using an exponential decay of the free plasma concentration from $c_{max}$ to $c_{trough}$ we then exploited the fact that the potency of an exponential function gives another factor in the exponential $\left( e^{-kt}\right) ^{hill} = e^{-kt*hill}$.

The derivation followed the line of subsection 3.3 and we finally obtained,

31 $$\begin{aligned} dose_{stasis}= & {} \, \, MEF\left( PTR, g/d, hill\right) \, \frac{IC50}{AUC_{DN,ub}/ \tau } \, \end{aligned}$$

32 $$\begin{aligned}= & {} \, MEF\left( PTR, g/d, hill\right) \, \frac{IC50}{c_{average,DN,ub}} \end{aligned}$$

with

33 $$\begin{aligned} MEF\left( PTR, g/d, hill\right) \,= \,\frac{PTR-1}{ln\left( PTR\right) } \left[ \frac{PTR^{g*hill/d}-1}{PTR^{hill}-PTR^{g*hill/d}}\right] ^{1/hill} . \end{aligned}$$

**Fig. 5:**
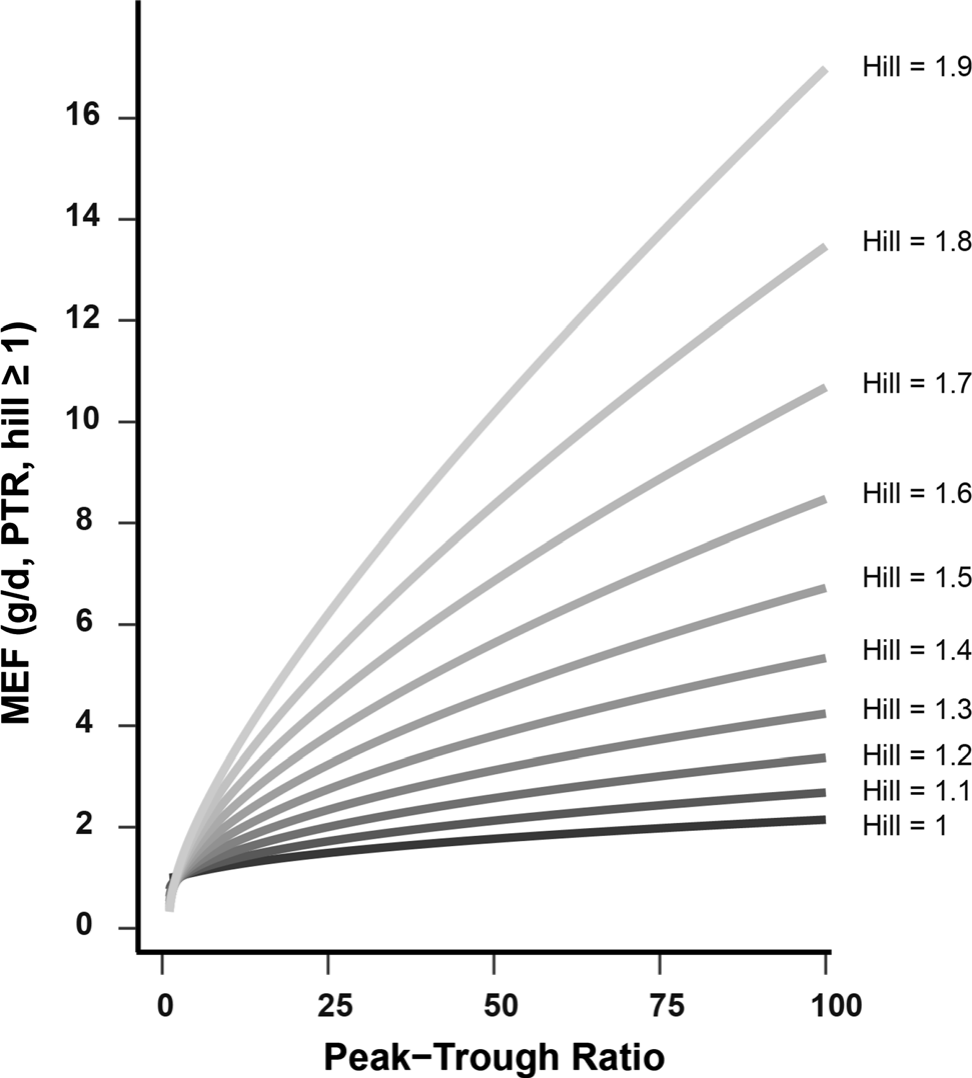
The model efficacy factor *MEF* which describes the necessary *IC*50 coverage for $c_{average, ub, stasis}$. We observed that the average concentration of the compound that is necessary to cover the *in-vitro IC50* for stasis becomes increasingly dependent on the *PTR* for Hill coefficients $>1$

Based on equation Eq. (33), we observed that the Hill coefficient appeared as an additional factor in the exponent of the *PTR*. Indeed, numerical simulation shown in Fig. (5) revealed that Hill coefficients $hill>1$ made $MEF\left( PTR, g/d, hill\right)$ of the necessary unbound average concentration (15), or AUC exposure (16), for tumour stasis, highly dependent on *PTR*.

These findings indicate that adjusting the *PTR* of a compound, while keeping its total exposure or average concentration constant, becomes more crucial in achieving an anti-tumor effect compared to merely changing the average concentration *per se*.

These observations can be easily understood. When compounds with the same average concentration have higher *PTR*s, their *IC*50 coverage in the anti-tumor response is less enduring according to the model in Eq. (30). Of note, this less enduring *IC*50 coverage can often not be compensated by higher $c_{max}$ values, once the Hill function Eq. (7) of the pharmacodynamic effect is saturated.^10^

This *PTR*-dependent response effect is even more pronounced for higher Hill coefficients, which correspond to steeper logistic curves. Consequently, the *PTR* and the elimination half-time under stasis conditions become increasingly critical for the anti-tumor effect when drug co-operativity is increased (hill > 1).

We finally note that the argument of Sect. 3.5.1 remained valid and, hence, assuming a separate absorption and elimination did not change our results, and both relaxations were combinable (the effect of the absorption phase is accounted for in the *PTR*).

### Understanding variability in the IVIVC by semi-mechanistic modelling

Using the model efficacy factor *MEF*(*PTR*, *g*/*d*) and the results of Fig. (5), we now can aim to explain a source of variability in the empirical relation between TGI, free *IC*50, and free average concentration.

**Fig. 6:**
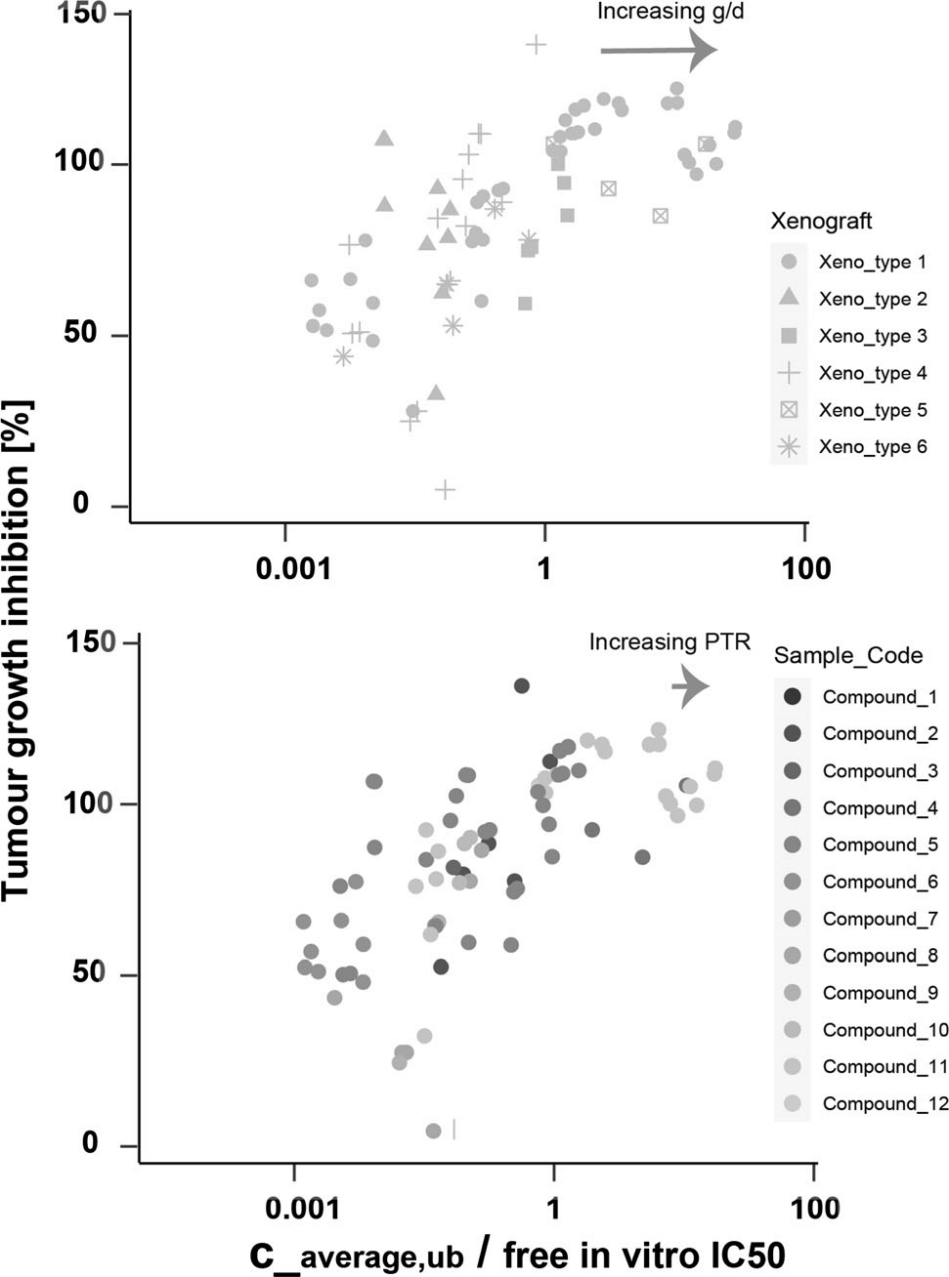
Variability in the empirical relation between TGI, free *IC*50 and free average concentration for the data set of Fig. 1 stratified according different xenograft types (having different *g*/*d* ratios) and different compounds (different *PTRs*; NB: compound *IC*50s are accounted for in the abscissa)

Looking specifically into our data set, we observed higher variability at stasis conditions (TGI=100) for different xenografts (upper graph) than for different compounds (lower graph) as seen in Fig. (6). These findings were consistent with our theoretical analysis above that the *g*/*d* ratio was more important than the *PTR* for the necessary *IC*50 coverage at tumor stasis.

However, since certain compounds were preferably studied in specific xenografts, both parameters were not completely independently studied, and a thorough analysis of the influence of *PTR* and *g*/*d* awaits further experimental studies.

### Extension of the method for studying efficacy in clinical populations

We finally sketch how our IVIVC considerations may be applied for studying drug efficacy in human populations, such as informing dosage decisions in clinical trials.^11^

We therefore assumed that each human tumour may be characterised by a certain *IC*50, and certain growth and decay rates. We first assumed that we can obtain a distributions of free *IC*50 from an *in-vitro* tumour cell panel that reflects the sensitivity of individual tumour cells to a specific compound (in a patient population and/or within an individual tumour).

Furthermore, we assumed that *g*/*d* rates of individual human tumours can be estimated from clinical data such as using longitudinal PET scans of tumours treated with compounds with comparable mode of action. As example, such longitudinal PET scan data of patient tumours are provided by consortia like the *Project Data Sphere* Initiative [22, 23].

Postulating that growth and decay rates are *in-vivo* specific parameters independent from the compound‘s *in-vitro* pharmacology (as in the pre-clinical case), we hence used above derivations for a human clinical dose estimation.

Replacing now single point estimates for *IC*50 and *g*/*d* with statistical variables $\left[ \textbf{IC50}\right]$ and $\left[ \mathbf{g/d}\right]$, we obtained^12^

34 $$\begin{aligned} \textbf{dose}_{\textbf{human}} = \tau \,\cdot \, \frac{PTR-1}{ln\left( PTR\right) } \frac{PTR^{ \left[ \mathbf{g/d}\right] }-1}{PTR-PTR^{\left[ \mathbf{g/d}\right] }} \frac{\left[ \textbf{IC50}\right] }{AUC_{DN,ub}} \, . \end{aligned}$$

With this, we obtained with ${\textbf{dose}_\textbf{human}}$ a dose distribution to treat a population of individual patients. As can easily been seen from Eq. (34), no tumour regression can be seen for patients with $g\ge d$ (no positive doses). Notably, from Eq. (34) we inferred that, if the statistical distributions of tumour properties $\textbf{g}$ and $\textbf{d}$ of a given population have same medians, the maximum population response would be limited to 50%, independent of the compound’s potency.

We hence suggest that this formalism can describe a *tumour idiosyncratic treatment resistance* mechanism that is wired in the individual *in-vivo* tumour growth and decay rates and that cannot be overcome by increased dosing.

### Discussion

#### General learnings

This paper presents a theoretical analysis of the Mayneord-like model, thereby investigating the drug-induced response of tumours and its connection to tumour stasis. Through our analysis, we have confirmed that the model framework is in line with an empirical *in-vivo* to *in-vitro* correlation (IVIVC). This correlation relates the free plasma concentration or exposure of a specific compound and its corresponding free *in-vitro* IC50 to the inhibition of tumour growth (TGI) using a simple formula [3, 4].

Specifically, we have shown that under the assumption of linear pharmacokinetics and the free plasma drug hypothesis, tumour stasis is essentially driven by the *IC*50 coverage of the unbound plasma drug concentration $c_{av, ub} / IC50_{ub}$ (or, equivalently, coverage of time integrated exposure $AUC_{ub}/IC50_{ub}$), the compound’s *PTR*, and the ratio *g*/*d* between the xenograft-specific tumour growth and decay rate. Our results will have impact on selecting appropriate xenograft models for proper clinical translation and on the understanding of how co-operativity of drug actions ($hill>$1) can determine $c_{max}$, $c_{trough}$ and $c_{average}$-drivenness.

Several further assumptions were made and the following specific conclusions were derived.

#### The mayneord-like model assuming linear tumour growth

Our analysis is centered around the unique characteristic of the Mayneord-like model, which assumes linear tumour growth over time. This model was derived from *in-vivo* analysis involving whole tumour resection in Jensen’s rat sarcomas, comparing untreated tissue (control) with tissue treated with X-radiations [16]. The study revealed that tumours grow alongside a rim, characterized by a necrotic core that lacks proliferation. Therefore, mathematical analyses have confirmed the intuitive assumption of sub-exponential growth dynamics, specifically zero-order growth.

It is important to note that certain idealizations were made during the mathematical analysis. For instance, the assumption of an infinitesimally small tumour rim and a tumour that can be mapped on a spherical shape, which may not hold true in experimental settings. Factors such as tumour space limitations, increased tumour vascularization, and changes in immune activity can also influence the growth profile in various ways.

Nonetheless, the Mayneord-like model has proven to be valuable in studying pre-clinical and clinical tumour growth over the past decade [8, 24]. As similar approaches, researchers such as Wang and colleagues, have utilized nonlinear mixed effect models with linear growth (and exponential decay) to describe response data in non-small-cell lung cancer patients [25]. Additionally, other models have been developed based on the assumption of sub-exponential tumour growth over time [26]. However, since exponential tumour growth, specifically when relatively slow (as observed in pre-clinical studies and slower-growing human tumours), can always be approximated using Taylor series expansion of tumour volume or radius, our analysis provides a reasonable approximation. Certainly, further experimental studies in pre-clinical and clinical settings that incorporate imaging and histology would be beneficial for understanding tumour growth dynamics in more detail.

A second specific feature of the Mayneord-like model is the distinction between *in-vitro* pharmacology (defined by the Hill function with an IC50 value determining the inhibition of cell proliferation) and *in-vivo*-specific tumour growth and decay rates. These factors establish a connection between the *in-vitro* pharmacology of a specific compound and *in-vivo* tumour growth.

While these factors are specific to a particular xenograft model, they are generally assumed to be mostly independent of the compound’s pharmacological profile, at least within a compound class with a similar mode of action. Various such *in-vivo* characteristics that cannot be captured by *in-vitro* cell assays include *in-vivo* tumour growth/aggressiveness, tumour drug exclusion, tumour cell-stroma interaction, and immune modulation in the presence and absence of treatment. Condensing these effects into just two parameters is undoubtedly an oversimplification, albeit one that has proven to be frequently useful in our pre-clinical research.

#### Pharmacokinetics and posology assumptions

For our investigation, we have made certain assumptions regarding the pharmacokinetics (PK) of the compounds. Specifically, we have assumed dose-linear PK, where the drug concentration in the body increases proportionally with the dose. We have also considered a single daily dose and steady-state kinetics, which involve separate first-order absorption and elimination kinetics. While it is true that many compounds exhibit exposure-limited effects at certain doses, assuming dose-linear PK is not overly restrictive for our analysis purposes. In fact, it is common practice to pre-select compounds during the pre-clinical stage to cover a range of exposures with dose-linearity that can achieve the desired effects in various xenograft models.

In contrast, assumptions about the shape of the pharmacokinetics may be more restrictive. Pre-clinical and clinical pharmacokinetics are complex and often require systematic physiology-based pharmacokinetics modelling (PBPK modelling). This involves integrating several effective compartments (organs) and considering the complex topology of their interconnections, as well as individual metabolic parameters specific to mice or humans [27]. Even if we assume a simple one-compartment model with absorption, obtaining an exact analytical solution for our analysis would require integrating a Bateman function of pharmacokinetics. However, the sum of two terms in the Bateman function would make the Hill function of Eq. (7) too complex for an analytical solution to our problem.

However, in oncology projects, pharmacokinetic (PK) variations often tend to be smaller compared to variations in pharmacodynamic (PD) parameters. This means that the distribution of *IC*50 values (a measure of drug potency) and the variability of growth and decay rates across tumours (g/d) are potentially more important drivers than the PK variations introduced by our assumption. This smaller dependence of efficacy on PK variations together with the fact that absorption phase (typically 0.5-2 h) and elimination phase (4-8 h) for our compounds were well separated made this assumption of a simpler two-exponential kinetics model reasonable for the class of MAPK inhibitors under investigation and potentially other small molecule compounds.

We finally note that results can be directly applicable to multiple daily doses per day, as long as doses are given in regular intervals. Thereby, the parameter $\tau$ duration of the dosing interval has to be adapted.

#### Non-co-operativity in the mayneord-like model leads to AUC-driven effects

Based on the assumptions of dose-linear pharmacokinetics (PK), non-cooperativity in the Mayneord-like model (*hill* = 1), and a one-compartment PK model with timely separable absorption and elimination phases, our analysis has revealed that the effective dose for tumour stasis is proportional to the fraction of free, dose-normalized area under the curve (*AUC*) of the compound and the free *in-vitro* *IC*50.

An interesting finding was that the necessary *IC*50 coverage required for tumour stasis shows only mild dependence on the peak-to-trough-ratio ratio (*PTR*) of the compound and, consequently, on the shape of the dose-response curve. This implies that the relationship between dose and effect is primarily driven by the total free exposure (or average concentration) of the compound, which aligns with the concept of an "AUC-driven" effect. Furthermore, this effect appears to be largely independent of the choice of xenograft model and the specific properties of the compounds, as long as the assumptions of kinetics and non-cooperativity hold true. It is important to note that the xenograft growth-to-decay ratios (*g*/*d* values) should fall therefore within the typical range of 0.3 to 0.7.

As shown in Sect. 3.5.2 this relation, however, changes once we assume $hill\,\ge \,1$. Specifically, higher Hill coefficients lead to an increased influence of the compound’s *PTR*, and hence its terminal half-life on the IC50 coverage factor for achieving tumour stasis. Our results therefore argue for dose-fractionation studies (with same daily doses, but different posology) to assess potential $c_{max}/c_{trough}$-drivenness, specifically in such cases.

#### Analysing non-pharmacology mediated resistance

As stated above, a key feature of the Mayneord-like model is the separation of pharmacology-dependent (Hill function) and xenograft model-dependent (and pharmacology independent) *in-vivo* properties. Essentially, this effect is captured by the model-dependent factor *MEF*(*g*, *d*, *PTR*) whose influence of the compound is only given by its *PTR* and this influence is smaller for broad *PTR* variations compared to varying *g*/*d* ratios when using different xenografts. These results emphasise the importance of choosing the right xenograft model for clinical translation.

Importantly, the model-dependent factor *MEF* incorporates a limitation where tumour stasis cannot be achieved if the tumour growth parameter (*g*) is greater than the model-corresponding decay parameter (*d*). This implies the existence of an in-built *in-vivo* resistance mechanism, where certain xenograft models would not respond to treatment regardless of the *IC*50 coverage. A typical mechanism of this solely *vivo* effect, may be inaccessibility of tumour parts to the compound or resistance through tumour immune editing. This analysis is facilitated by the population formalism in Eq. (34), where we have distinguished between pharmacology resistance (ineffective *IC*50-coverage) and tumour idiosyncratic resistance ($g \ge d$).

This idea of tumour idiosyncratic resistance could be beneficial when transferring these modeling attempts to clinical scenarios. Specifically, we would obtain *g*/*d* ratios from longitudinal PET scans of individual patient tumors in clinical trials from dedicated repositories such as *Project Data Sphere* and integrate them into our model framework. Thereby, the developed model framework would enable mechanistic comprehension of potential resistance mechanisms. This type of clinical back-translation is ongoing in our group.

#### Applying findings to other therapeutic areas

Although the Mayneord-like model was designed for tumor growth, our results could be applicable to fields beyond oncology, where a pathological effect (here tumor growth) is counteracted by a compound-induced treatment. This generalization is valid as long as the treatment can be represented by a Hill function, possesses *in-vitro* efficacy parameters, and adheres to the pharmacokinetic assumptions mentioned earlier. Consequently, we can substitute our assumptions of pathological deviation and treatment with an alternative *in-vivo* efficacy model that includes specific clinical remodeling parameters, *in-vitro* pharmacological effects, and clinical outcomes tailored to the pathology under study.

## Glossary

**Table:** 

Symbol	Unit	Description
*AUC* *ub*	*nM* h	Unbound AUC of the compound
$AUC_{DN,ub}$	*nM* h/(mg/kg)	Dose normalised unbound AUC
$AUC_{effect}$	*h*	*effective AUC*
		effective duration of *IC*50 coverage by compound *in-vivo*
$c_{trough}, c_{max}, c_{average}$	*nM*	min, max and average conc. of the compound
$c_{trough, stasis}, c_{max, stasis}$	*nM*	min, max and average conc.
$c_{average, stasis}$	*nM*	of the compound required for tumour stasis (TGI=100 %)
$dose_{stasis}$	*mg*/*kg*	dose required for stasis
*d*	*mm*/*h*	tumour decay rate of a specific xenograft
*g*	*mm*/*h*	tumour growth rate of a specific xenograft
*hill*	none	Hill coefficient of the $E_{max}$ function, compound co-operativity
*IC*50	*nM*	*in-vitro* 50% inhibition of cell growth
$k_a$, $k_e$	$h^{-1}$	absorption and elimination rate constant
$MEF\left( PTR, g/d, Hill\right)$	none	*Model efficacy factor*
		factor defining the necessary *IC*50 coverage ratio for a compound
		characterised by *PTR* and *hill* and a xenograft by *g*/*d*
*PTR*	none	= $c_{max}/c_{trough}$, peak-trough ratio
*R*, $R_{0}$	*mm*	tumour radius, initial tumour radius
*TGI*	none	tumour growth inhibition
$TGI_{min}, TGI_{max}, PD_{inflex}$	none	minimum, maximum and inflection point of the empirical logistic fit
$\tau$	*h*	dosing interval
$V^{control}_{tumour}, V^{treated}_{tumour}$	$mm^3$	total tumour volume for control and treated tumours

### Supplementary Information

Below is the link to the electronic supplementary material.

Supplementary file1 (PDF 281 kb): An analysis of the inflection points of the IVIVC curves.

Supplementary file1 (XLSX 99 kb): Calculation of stasis doses for exposure, *PTR* and *g/d* ratios

Supplementary file1 (CSV 5 kb): Analysis for Appendix A.

## Figures and Tables

**Fig. 7:**
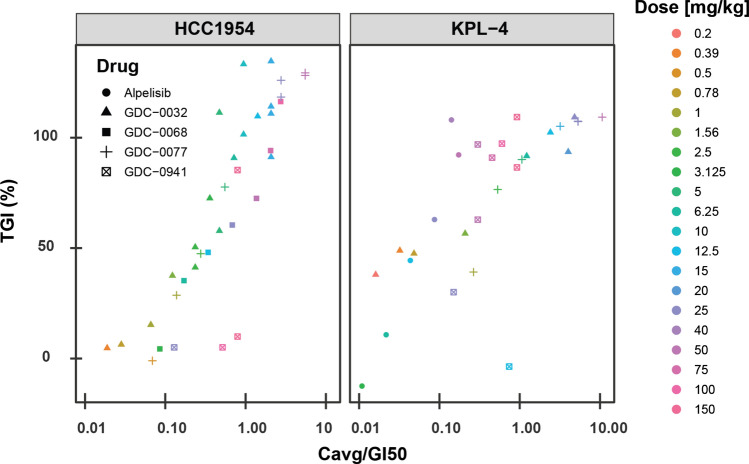
IVIVC relationship for the study [28] as reanalyzed by us. Shapes are different compounds, color indicate different doses. Fair alignment for studies with different doses an compounds within the same cell lines are observed, with a weak tendency shift between cell line data, consistent with our analysis Fig. 6

**Fig. 8:**
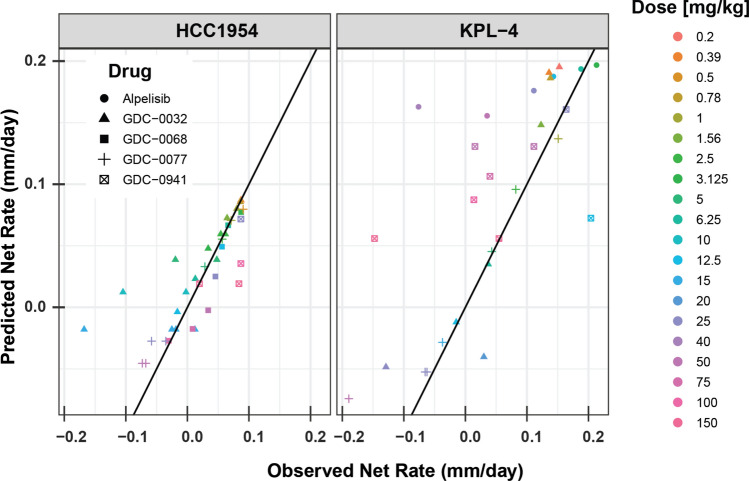
Remodelling of decay rate for the study [28]. Shapes represent various compounds, while colors signify different doses. Drug-induced tumor growth inhibition was re-modeled by assuming our model relation, Eq. 20, and considering the published drug concentration, as well as cell and compound-specific *in-vitro* IC50 values. Decay rates for each cell were optimized to best explain the published (observed) net rates (tumor growth minus decay rates, x-Axis) of the tumors following pharmacological intervention. Xenograft-specific *g*/*d* ratios were determined to be 0.56 and 0.67 for HCC1954 and KPL-4, respectively

## Appendix A illustration of separation of *in-vitro* and *in-vitro* effects in the PK/TGI model

The major goal of the decay rate *d* is to separate properties of the *in-vivo* tumour from the pharmacologic action the compound acts upon the cell. Specifically, as *in-vitro* dose response curves follow an Hill function, we separated the *in-vitro* effect explicitly. The decay rate *d* is hence a proportionality factor specific to the tumour and is assumed not to depend in good approximation on the drug and the concentration. As example, one would envision two tumours with different drug permeabilities due to different stroma content. This independency is the assumption of almost all modelling efforts for xenograft studies at Boehringer Ingelheim. We further illustrate this here by an external data set published by Genentech [28].

Our analysis of their data firstly demonstrates that a similar IVIVC analysis for their compounds can be given as for ours. It further demonstrates that these relations differ for the two used cell line grafts, while different compounds and different doses well align for HCC1954. Results also demonstrate a fair alignment between *IC*50 coverage and effect in the KPL-4 cell lines, with some inter-study variability, but no major deviations between different compounds.

Our subsequent goal was to examine the growth and decay rates for each cell line and to ascertain if our model assumption, Eq. (20), is applicable for all compounds and concentrations used in the study. We derived the growth rates for each cell line directly from the control data. To establish the decay rates of our model and to illustrate their independence from the compound and dose, we calculated the net tumor growth rates from the study data. This net rate was determined using the information provided on the relative change in rate between control and treated xenografts (Supplement Table 2).^13^ By applying our model, Eq. 20, we were able to achieve fits between the predicted and observed net rates (and consequently values of *d*). The quality of these fits (Fig. 7) demonstrated that these fits were more dependent on the cell lines than on specific compounds across doses, thereby validating our model assumption. Specifically, we found *g* and *d* values for HCC1954 to be 0.09 and 0.16 mm/day, and 0.20 and 0.30 mm/day for KPL-4 (*g*/*d* ratios of 0.56 and 0.67, respectively).

## Appendix B derivations of equations for stasis condition for negligible absorption

### B.1 derivation equation (23)

We calculate

B1 $$\begin{aligned} AUC_{effect} = \int ^{\tau }_0\, \frac{e^{-kt}}{\left( e^{-kt} + \frac{IC50}{c_{max}}\right) }\,dt \, , \end{aligned}$$

by substituting

B2 $$\begin{aligned} u(t)& = &{e^{-kt}}\,\rightarrow du=\, -k\, e^{-kt}\, dt\,\rightarrow \, dt = - \frac{1}{u\,k}\, du\, \end{aligned}$$

B3 $$\begin{aligned} u_0: = u(0)& = &1 \end{aligned}$$

B4 $$\begin{aligned} u_\tau : = u(\tau )& = &e^{-k\tau } = \frac{c_{trough}}{c_{max}} \end{aligned}$$

into Eq. (B1) gives

B5 $$\begin{aligned} AUC_{effect}& = &\int ^{u_\tau }_{u_0}\, \, \frac{u}{\left( u + \frac{IC50}{c_{max}}\right) }\,\left( \frac{-1}{uk}\right) \, du\, \end{aligned}$$

B6 $$\begin{aligned}& = &\int ^{u_0}_{u_\tau }\, \, \frac{1}{k\,\left( u + \frac{IC50}{c_{max}}\right) }\, du\, \end{aligned}$$

B7 $$\begin{aligned}& = &\frac{1}{k}\, \left[ ln\left( u\,+ \frac{IC50}{c_{max}} \right) \right] \Bigr \Vert _{ \frac{c_{trough}}{c_{max}} }^{1} \end{aligned}$$

B8 $$\begin{aligned}& = &\frac{\tau }{\text {ln}\left( PTR\right) }\left[ \text {ln}\left( 1+ \frac{IC50}{c_{max}} \right) - \text {ln}\left( \frac{c_{trough}}{c_{max}}\,+ \frac{IC50}{c_{max}} \right) \right] \, \end{aligned}$$

B9 $$\begin{aligned}& = &\frac{\tau }{\text {ln}\left( PTR\right) }\text {ln}\left[ \frac{c_{max} + IC50}{c_{trough} + IC50} \right] . \end{aligned}$$

### B.2 Derivation Equation (26)

We are first setting Eq. (B9) in $AUC_{effect}$ into the condition for stasis Eq. (24)

10 $$\begin{aligned} ln\left( \frac{c_{max} + IC50}{c_{trough} + IC50} \right) \,\frac{\tau }{ln\left( PTR \right) }=\, \frac{g}{d}\cdot \tau . \end{aligned}$$

and, hence

11 $$\begin{aligned} ln\left( \frac{c_{max} + IC50}{c_{trough} + IC50} \right) = \frac{g}{d}\, ln\left( PTR \right) . \end{aligned}$$

We now take the exponentials on both sides

12 $$\begin{aligned} \frac{c_{max} + IC50}{c_{trough} + IC50}& = &exp\left( \frac{g}{d}*ln\left( PTR \right) \right) \nonumber \\ \frac{PTR*c_{trough} + IC50}{c_{trough} + IC50}& = &exp\left( (ln\left( PTR \right) ^{g/d}\right) \nonumber \\& = &PTR^{g/d}\nonumber \\ PTR\,c_{trough} +\, IC50& = &PTR^{g/d}\,\left( c_{trough} +\, IC50\right) \end{aligned}$$

we get the following conditions for tumour stasis from Eq. (11)

13 $$\begin{aligned} c_{trough, stasis, ub} = IC50 \frac{PTR^{g/d}-1}{PTR-PTR^{g/d}} \end{aligned}$$

By similar calculations, we obtain

14 $$\begin{aligned} c_{max, stasis, ub} = IC50\,\cdot \, PTR \frac{PTR^{g/d}-1}{PTR-PTR^{g/d}} \end{aligned}$$

and with

15 $$\begin{aligned} c_{average}& = &\frac{1}{\tau }\int ^{\tau }_{0}\,c_{plasma, \,free}(t)\,dt \,= \frac{1}{\tau }\int ^{\tau }_{0}\, c_{max}\,e^{-kt}\,dt\nonumber \\& = &\, \frac{ c_{max}- c_{trough}}{ ln\left( PTR \right) }, \end{aligned}$$

wet get the necessary average concentration for tumour stasis.

16 $$\begin{aligned} c_{average, stasis, ub}& = &\,\frac{\left( c_{max}\,-c_{trough}\right) }{ln\left( PTR\right) }\nonumber \\& = &\,\frac{c_{trough}\,\left( PTR\,-1\right) }{ln\left( PTR\right) }\nonumber \\& = &IC50\,\cdot \, \frac{PTR-1}{ln\left( PTR\right) } \frac{PTR^{g/d}-1}{PTR-PTR^{g/d}}. \end{aligned}$$

## Derivation of $AUC_{effect}$ for non-negligible fast absorption

We follow the assumption of a one-compartment model with an absorption rate $k_a$ and an elimination rate $k_e$ which gives the well-known Bateman function for a single exposure.

Provided that $k_a$ and $k_e$ are well distinct (which they usually are for most oncology compounds), both increase during the absorption phase and decrease in the elimination phase can be considered as exponential, yet at different speed^14^. We therefore can split the expression of our effective AUC ($AUC_{effect}$) calculation of (B9). Thus, we can write

17 $$\begin{aligned} AUC_{effect}\left( total\right)& = &AUC_{effect}\left( trough\rightarrow peak\right) \Bigr \Vert _{0}^{t_{max}}\,\nonumber \\& + &AUC_{effect}\left( peak\rightarrow trough \right) \Bigr \Vert _{t_{max}}^{\tau } . \end{aligned}$$

The second term is essentially the calculated ($AUC_{effect}$) of (B9) within shorter boundaries and modified $k_e$,

18 $$\begin{aligned} AUC_{effect}\left( peak\rightarrow trough \right) \Bigr \Vert _{t_{max}}^{\tau }\,= & {} \,\frac{\tau - t_{max}}{ln\left( PTR\right) }\, ln\left[ \frac{c_{max} + IC50}{c_{trough} + IC50} \right] \end{aligned}$$

19 $$\begin{aligned} k_e \,= & {} \, \frac{1}{\tau -t_{max}}\,ln\left( \frac{c_{trough}}{c_{max}} \right) = \frac{1}{\tau -t_{max}}\,\left( \frac{c_{trough}}{c_{max}}\right) . \end{aligned}$$

The first terms needs a bit attention,

20 $$\begin{aligned} AUC_{effect}{} & {} \left( trough\rightarrow peak\right) \Bigr \Vert _{0}^{t_{max}}\, =\,\int ^{t_{max}}_0\, \frac{e^{k_a t}\cdot c_{trough}}{\left( e^{k_a t}\cdot c_{trough} + {IC50}\right) }\,dt \nonumber \\& = & \int _{t_{max}}^0\, \frac{e^{k_a \left( t_{max}-t' \right) }\cdot c_{trough}}{\left( e^{k_a \left( t_{max}-t' \right) }\cdot c_{trough} + {IC50}\right) }\,\left( -dt\prime \right) \nonumber \\& = & - \int _{t_{max}}^0\, \frac{e^{-k_a t\prime }\cdot c_{max}}{\left( e^{-k_a t\prime }\cdot c_{max} + {IC50}\right) }\,dt\prime \nonumber \\& = & \int ^{t_{max}}_0\, \frac{e^{-k_a t\prime }}{e^{-k_a t\prime } + \frac{IC50}{c_{max}}}\,dt\prime \, \nonumber \\& = &\frac{t_{max}}{ln\left( PTR\right) }\, ln\left[ \frac{c_{max} + IC50}{c_{trough} + IC50} \right] \,, \end{aligned}$$

where we have formally changed the direction of integration from $t\prime \rightarrow t_{max} -t$ and hence from $c_{max}\rightarrow c_{trough}$. We thereby further used the relations

21 $$\begin{aligned} c_{max}& = &c_{trough}*e^{k_a t_{max}} \end{aligned}$$

22 $$\begin{aligned} k_a& = &\frac{1}{t_{max}}\,ln\left( \frac{c_{max}}{c_{trough}}\right) . \end{aligned}$$

By adding up the terms Eqs. (18) and (20) in Eq. (17), we see that this result in the same term as (B9). We therefore conclude that our reasoning can be extended to situations with non-negligible absorption when both phases can be separated and the PK dosing curve along the dosing interval is at steady state.

The independence of the integrated response (*AUC* or $AUC_{effect}$) from the duration of the absorption phase (given the limitations below) can intuitively understood. Specifically, while non-negligible absorption reduces the maximum free concentration $c_{max}$, this maximum is shifted to later time points which results in a higher $c_{trough}$ due to shorter decay period and assuming steady state.

We note that a similar argument can be made for the calculation of the average concentration as of Eq. (15), which is needed to render the analysis of the previous section correct also for non-negligible absorption phase.

23 $$\begin{aligned} c_{average} \,= & {} \, \frac{1}{\tau }\left[ AUC\,\left( trough\rightarrow peak\right) \Bigr \Vert _{0}^{t_{max}} + \,AUC\,\left( peak\rightarrow trough \right) \Bigr \Vert _{t_{max}}^{\tau }\right] \nonumber \\ \,= & {} \,\frac{1}{\tau }\left( \int ^{t_{max}}_{0}\,c_{trough}\,e^{k_a t}\,dt + \int ^{\tau }_{t_{max}}\,c_{max}\,e^{-k_e t} dt\right) \end{aligned}$$

Analogous to Eq. (15), the second integral gives

24 $$\begin{aligned} \int ^{\tau }_{t_{max}}\,c_{max}\,e^{-k_e t} dt \,= & {} \,-\frac{ c_{trough}- c_{max}}{k_e}\nonumber \\ \,= & {} \,\frac{\tau -t_{max}}{ln\left( PTR\right) }\left( c_{max}- c_{trough}\right) \,, \end{aligned}$$

while we can again apply symmetry relations $t\prime \rightarrow t_{max}-t$ for the first integral

25 $$\begin{aligned} \int ^{ t_{max}}_{0}\,c_{trough}\,e^{k_a t}\,dt\,= & {} \,\int ^{0}_{ t_{max}}\,c_{trough} \,e^{k_a (t_{max}-t\prime ))}\,(-dt \prime ) \nonumber \\ \,= & {} \,\int ^{0}_{ t_{max}}\,c_{max} e^{-k_a t\prime }(-dt \prime ) \nonumber \\ \,= & {} \,\int ^{t_{max}}_{ 0}\,c_{max} e^{-k_a t\prime }\,dt \prime \nonumber \\ \,= & {} \,-\frac{c_{trough}-c_{max}}{k_a} \nonumber \\ \,= & {} t_{max}\,\frac{c_{max}-c_{trough}}{ln\left( PTR\right) } \end{aligned}$$

and hence summarising both integrals in Eq. (23)

26 $$\begin{aligned} c_{average} = \frac{c_{max}-c_{trough}}{ln\left( PTR\right) } \end{aligned}$$

## Footnotes

1 Some models use exponential volume growth over time. We note that in case of slow growth, these models may be equivalent to ours since a Taylor-series expansion can be assumed. Moreover, specifically under treatment, tumour growth is slow and proliferation may hence be still in the first order regime.

2 We would like to emphasise here, that also the *in-vitro* *IC*50 is specific of the tumour cell graft model used (and is accordingly measured in *in-vitro* assays). However, to distinguish this xenograft specific feature from those that emerge in 3D *in-vivo* tumours of a specific xenograft type (characterised by *g* and *d*), we will denote the latter as *in-vivo specific xenograft properties* in the text.

3 One of the motivations is as follows: Consider an idealized scenario where a set of compounds binds to a target with varying degrees of inhibition strength, assuming the same target engagement characteristics and mode of action. Additionally, assume that these compounds have no off-target effects. In such a scenario, any response curve obtained using this set of compounds at identical doses or exposures would yield the same pharmacological response curves as using a single compound at different doses.

4 Here, the *IC*50 is defined as the concentration of the drug that inhibits 50% proliferation of tumor cells in an *in-vitro* experiment. Sometimes, this factor is also referred to as EC50 to differentiate it from the biochemical inhibitory capacity (*IC*50) of the drug towards the target. However, for the sake of consistency, we use *IC*50 in the aforementioned sense in this paper. It is also worth noting that these experiments are typically conducted in the presence of fetal calf serum and therefore need to be adjusted for plasma protein binding (free *IC*50). For simplicity, we consider the *IC*50 as the free *IC*50 throughout the paper.

5 It is important to note that our analysis does not aim to specify exact dose units (such as nM or mg/kg), as we seek to maintain the generality of our conclusions. Assuming that average unbound concentrations and *IC*50 are both expressed in concentration units of the same scale, the coverage factor would have units of inverse dose and inverse dose multiplied by time, respectively. Yet, our derivations remain valid as long as the dose and dose normalization on both sides of the equations use the same scaling units throughout the text, and as long as *IC*50 and $c_{average, DN,ub}$ are expressed in the same concentration units.

6 We note that, while, as can be seen from Fig. (2), steady state may not have been reached yet, Eq. (20) still provides a sufficient boundary criterion for tumour stasis (with the equal sign indicating the borderline condition for stasis).

7 We note that for constant concentrations $c_{max}\,=c_{trough} = c_{const}$   numerator and denominator in Eq. (16) will converge against 0. Following the rule of de l’Hospital, it can easily be shown that $\lim _{c_{max}\rightarrow c_{trough}}\, c_{average} = c_{const}$.

8 This is an analogy to the discovery of the Hill function where co-operativity between oxygen-binding sites in hemoglobin was observed, such that binding of one oxygen molecule facilitated the binding of others.

9 For other compounds with a one compartment model with first-order absorption, a Bateman function would be needed to be used [21], which would severely aggravate the mathematical analysis.

10 Please note that that our analysis is designed to explore effective doses to exactly cover tumor stasis. Therefore, we expect *IC*50 to be centred between (or at least be close to) $c_{max}$ and $c_{trough}$. Then, increasing *PTR* and maintaining same exposure, shifts both PK extrema away from the *IC*50 coverage line in opposite directions. Consequently, compounds with high *PTR* are very likely to have *"unproductive AUC"* due to saturation of the Hill PD curve.

11 For purpose of the argument, we again assumed the special case of *hill*=1.

12 We deliberately assumed point estimations for and *PTR* dose-normalised $AUC_{DN,ub}$ as they are characteristic for the compound under investigation and, hence, their variability can be managed compared to those in $\left[ IC50\right]$ and $\left[ g/d\right]$.
