# Supplementary material for: Explaining in-vitro to in-vivo efficacy correlations in oncology pre-clinical development via a semi-mechanistic mathematical model
Source: J Pharmacokinet Pharmacodyn. 2023 Nov 6;51(2):169–85. doi: 10.1007/s10928-023-09891-7 (PMC10982099; doi:10.1007/s10928-023-09891-7)
Supplement: Supplementary file 1 — Supplementary file1 (PDF 281 kb): An analysis of the inflection points of the IVIVC curves. [file 10928_2023_9891_MOESM1_ESM.pdf]

Supplement: Explaining *in-vitro* to *in-vivo* efficacy correlations in oncology pre-clinical development via a semi-mechanistic mathematical model

Heinrich J. Huber[1*] and Hitesh B. Mistry[2]

[1*]Drug Discovery Sciences, Boehringer Ingelheim RCV GmbH & Co KG, Dr. Boehringer-Gasse 5-11, Vienna, 1120, Austria.
[2]Department, SEDA Pharmaceutical Development Services, Oakfield Road Cheadle Royal Business Park, Cheadle, SK8 3GX, United Kingdom.

*Corresponding author(s). E-mail(s): heinrich_2.huber@boehringer-ingelheim.com;
Contributing authors: hitesh.mistry@sedapds.com;

1 Assessing IVIVC at the inflection point

In subsection 3.2 of the main text we have given a formula for xenograft-specific assessment for evaluating tumour stasis. We now were interested in assessing the xenograft specific point of inflection of the IVIVC curve $PD_{inflex,g,d}$. Specifically, we are looking for ($IC50$-normalised) exposure that defines this point of inflection for the parameter set g, d and PTR. We therefore assume that all studies that are covered in our in-vitro to in-vivo correlation had the same treatment cycles (not doses). We further assumed that studies were assessed at similar days, such that maximum possible efficacy was achieved and no resistances were yet developed.

Following the definition of the inflection point we have,

$$x = arg_x\left(TGI = 50\right) = PD_{inflex,g,d,PTR} = \frac{c_{average,ub}}{IC50_{ub}}. \tag{1}$$

We first start from the definition of the TGI (Eq. 9 in main) and evaluate its dependency on the coverage factor x. We note that only $V_\tau := V_{tumour}^{treated}(end = \tau)$ is dependent on x. We further assume n dosage cycles close to the steady state and get:

$$\frac{d^2}{dx^2}\left(TGI\left(x\right)\right) = 0 \rightarrow \frac{d^2}{dx}\left(V_{tumour}^{treated}(end = \tau, x)\right) = 0, \tag{2}$$

$$\frac{d^2}{dx^2} V_\tau\left(x\right) = \frac{d^2}{dx}\left(\frac{4\pi}{3} R_\tau\left(x\right)^3\right). \tag{3}$$

Using the dot notation for the derivative according X, we obtain thee second derivative of $\ddot{V}_\tau$,

$$\begin{aligned}
\ddot{V}_\tau &= 4\pi\left[2\, R_\tau\, \dot{R}_\tau^2 + R_\tau^2 \cdot \ddot{R}_\tau\right] = 0,\\
0 &= 2\,\dot{R}^2 + \ddot{R}\cdot R,\\
0 &= 2\,d^2\, \cdot n^2 \dot{A}UC^2 - d\cdot n\cdot\, \ddot{A}UC\left(R_0 + g - d\,AUC\right),
\end{aligned} \tag{4}$$

where we have omitted the index τ for brevity and assumed $R \neq 0$. We remember the definition of the radius in Eq. (M.20) main with the definition of the effective AUC, AUC_{effect} Main Eq. (M.24).

$$\begin{aligned}
\bar{A}UC &= \frac{\tau}{ln\left(PTR\right)}\, ln\left(\frac{c_{max} + IC50}{c_{trough} + IC50}\right)\\
\bar{A}UC &= \frac{\tau}{ln\left(PTR\right)}\, ln\left(\frac{y + 1}{r\cdot y + 1}\right)\\
\text{with} &= y = \frac{c_{max}}{IC50} \quad \text{and} \quad r = \frac{1}{PTR}.
\end{aligned} \tag{5}$$

Instead of $x = \frac{c_{average}}{IC50}$ above AUC_{effect} is dependent on $y = \frac{c_{max}}{IC50}$. To transform the variable, we can avail of Eq. (M.22). Since for the pre-clinical case, the peak-through ratios are high, we can neglect c_{trough} and replace

$$c_{average} = \frac{c_{max}}{ln\left(PTR\right)} \quad \rightarrow \quad y = x\cdot ln\left(PTR\right) \quad \rightarrow \quad dy = dx\cdot ln\left(PTR\right) \tag{6}$$

where the peak-through ratio was assumed as constant for a compound as a consequence of linear dosing. We now can calculate the derivatives

$$\begin{aligned}
\dot{A}UC &= \frac{d}{dx}\bar{A}UC = \frac{d}{dy}\frac{dy}{dx}\bar{A}UC = \frac{dy}{dx}\frac{d}{dy}\bar{A}UC = ln\left(PTR\right)\frac{d}{dy}\bar{A}UC\\
&= ln\left(PTR\right)\cdot\frac{d}{dy}\left[\frac{\tau}{ln\left(PTR\right)}\cdot ln\left(\frac{y + 1}{r\cdot y + 1}\right)\right]\\
&= \tau\frac{d}{dy}\left[ln\left(y + 1\right) - ln\left(ry + 1\right)\right]
\end{aligned}$$

$$
\begin{aligned}
&= \tau \left[\frac{1}{y+1} - \frac{r}{ry+1} \right] \\
&= \tau \frac{1-r}{(y+1)\,(ry+1)}\,, \qquad (7)
\end{aligned}
$$

and

$$
\begin{aligned}
\ddot{AUC} &= \frac{d}{dx}\,\dot{AUC} = ln\,(PTR)\,\frac{d}{dy}\,\bar{AUC} \qquad (8) \\
&= ln\,(PTR)\,\frac{d}{dy}\left[\tau\left[\frac{1}{y+1} - \frac{r}{ry+1}\right]\right] \\
&= \tau \cdot ln\,(PTR)\left[-\frac{1}{(y+1)^2} + \frac{r^2}{(ry+1)^2}\right] \\
&= \tau \cdot ln\,(PTR) \cdot \frac{(1-r)\,(1+r+2yr)}{(y+1)^2\,(ry+1)^2}\,. \qquad (9)
\end{aligned}
$$

We now set the derivatives Eq. (7) and (9) into the condition for the inflection point (4) while using the definition of the radius Eq. (**??**),

$$
\begin{aligned}
0 &= 2\,d^2 \cdot n^2 \cdot \dot{AUC}^2 - d \cdot n \cdot \ddot{AUC}\,\left(R_0 + g - d\,AUC\right) \\
0 &= -2\,d^2 \cdot n^2 \cdot \tau^2 \cdot \left(\frac{1-r}{(y+1)\,(ry+1)}\right)^2 \\
&+ d \cdot \tau \cdot \left(R_0 + g\tau - d \cdot n \cdot \bar{AUC}\right) \cdot ln\,(PTR) \cdot \left(\frac{(1-r)\,(1+r+2yr)}{(y+1)^2\,(ry+1)^2}\right) \\
0 &= -2\,d \cdot n\tau^2 \cdot (1-r) \\
&+ \tau\,n\,ln\,(PTR)\,(1+r+2yr)\left(R_0 + g\tau - d \cdot n\,\frac{\tau}{ln\,(PTR)} \cdot ln\left(\frac{y+1}{r \cdot y+1}\right)\right) \qquad (10)
\end{aligned}
$$

We finally make an assumptions of a high peak-through ratio for compounds in a pre-clinical setting ($PTR \approx 40 - 150$) and $c_{trough} \ll IC50$ (confer Fig. **??** for validity). Specifically, we assume

$$
r = \frac{1}{PTR} \ll 1 \quad \text{and} \quad ry = \frac{c_{trough}}{IC50} \ll 1 \qquad (11)
$$

and we get

$$
2 \cdot d + ln\,(PTR)\left(\frac{R_0}{(\cdot n\tau)} + g\right) - d \cdot ln\,(y+1) = 0, \qquad (12)
$$

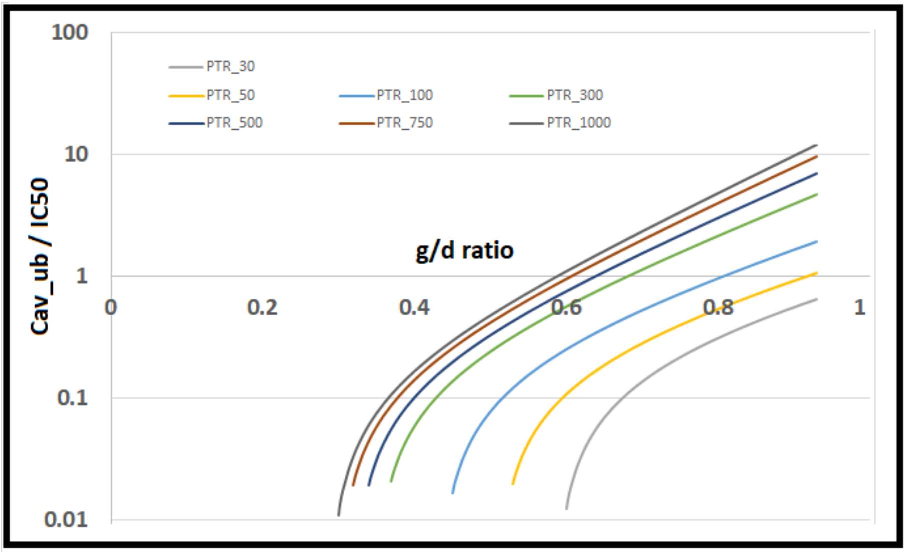

Fig. 1 Calculation of the inflection point (TGI = 50%) for different g/d and peak-through ratios PTR. A model breakdown for fast growing and highly treatment- sensitive tumours is observed. In such tumours, we would expect a shift of curves to lower x-values by replacing g/d by $g + R_0/\tau/d$

and finally by re-subsituting $y = ln\,(PTR)\;x$

$$x = \frac{1}{ln\,(PTR)} \cdot \left(e^{-2+\left(\frac{ln(PTR)(R_0/(\cdot n\tau)+g)}{d}\right)} - 1 \right) . \qquad (13)$$

Thereby, we have obtained the value $x = PD_{inflex} = c_{average}/IC50$ for the inflection point. Assuming a small initial radius R_0 and a large interval, we can assume $g \gg \frac{R_0}{n\,\tau}$, and would obtain

$$x = \frac{1}{ln\,(PTR)} \cdot \left(e^{-2} \cdot (PTR)^{g/d} - 1 \right) . \qquad (14)$$

The point of inflections $x = PD_{inflex} = c_{av_i nflex}/IC50$ are shown in Fig (1) for different peak-through ratios PTR. As we can see that the simplified model breaks down for slow growing and highly treatment-sensitive xenograft models (leading to $x < 0$). We note that in such tumours, the initial tumour radius R_0 cannot anymore neglected. Indeed, the underlying assumption often breaks down for our experimental studies with $R_0 \approx 3$ cm, $g \approx 1.E - 2$ mm/h, $\tau =$ 24h and $n = 14$ dosage cycles. For such cases, we have to suffice with Eq. (13).

2 Discussion of Analysis of the inflection point of the IVIVC curve

We investigated the analytical relationship between the PK and model parameters as model inputs and the inflection point of the $IC50$ coverage factor $(AUC_{ub}/IC50_{ub})$ with respect to tumour growth inhibition. This point is defined by the change of dynamics, whereby increase in exposure compared to the in-vitro $IC50$ becomes successively less important until a final saturation is achieved. This point can also be seen as such exposure, where variations in exposure translate into high variations in TGI (i.e. the variability of exposure, considered as first derivative of the TGI is maximum). Hence, exposure variations at this point are highly relevant for assessing the effect of PK fluctuations. Estimating this point analytically, may also serve for study sub maximum effects as they are desired for exploring drug combinations.

Setting the second derivative of the TGI function with respect to $c_{av,ub}/IC50_{ub}$ to zero, indeed led to an analytical relationship allowing us to determine the appropriate exposure values. However, to our dismay, results in Eq. (13) suggest that this exposure was dependent on the initial tumour radius R_0 which is often dependent on the individual study, aggravating general conclusions. We therefore made an approximation Eq. (14) which, however, based on calculations with our compounds (results not shown) would often not hold true. Hence, further work and assumptions may be needed for an exact determination of this point.
